# Pharmaceutical care issues identified by pharmacists in patients with diabetes, hypertension or hyperlipidaemia in primary care settings

**DOI:** 10.1186/1472-6963-12-388

**Published:** 2012-11-12

**Authors:** Siew Siang Chua, Li Ching Kok, Faridah Aryani Md Yusof, Guang Hui Tang, Shaun Wen Huey Lee, Benny Efendie, Thomas Paraidathathu

**Affiliations:** 1 Department of Pharmacy, Faculty of Medicine, University of Malaya, Kuala Lumpur, Malaysia; 2 Clinical Research Centre, Kuala Lumpur Hospital, Kuala Lumpur, Malaysia; 3 Faculty of Pharmacy & Allied Health Sciences, SEGI University College, Selangor, Malaysia; 4 School of Pharmacy and Health Sciences, International Medical University, Kuala Lumpur, Malaysia; 5 Faculty of Pharmacy, Universiti Kebangsaan Malaysia, Kuala Lumpur, Malaysia

**Keywords:** Pharmaceutical care, Pharmacist, Chronic disease, Intervention, Drug-related problem

## Abstract

**Background:**

The roles of pharmacists have evolved from product oriented, dispensing of medications to more patient-focused services such as the provision of pharmaceutical care. Such pharmacy service is also becoming more widely practised in Malaysia but is not well documented. Therefore, this study is warranted to fill this information gap by identifying the types of pharmaceutical care issues (PCIs) encountered by primary care patients with diabetes mellitus, hypertension or hyperlipidaemia in Malaysia.

**Methods:**

This study was part of a large controlled trial that evaluated the outcomes of multiprofessional collaboration which involved medical general practitioners, pharmacists, dietitians and nurses in managing diabetes mellitus, hypertension and hyperlipidaemia in primary care settings. A total of 477 patients were recruited by 44 general practitioners in the Klang Valley. These patients were counselled by the various healthcare professionals and followed-up for 6 months.

**Results:**

Of the 477 participants, 53.7% had at least one PCI, with a total of 706 PCIs. These included drug-use problems (33.3%), insufficient awareness and knowledge about disease condition and medication (20.4%), adverse drug reactions (15.6%), therapeutic failure (13.9%), drug-choice problems (9.5%) and dosing problems (3.4%). Non-adherence to medications topped the list of drug-use problems, followed by incorrect administration of medications. More than half of the PCIs (52%) were classified as probably clinically insignificant, 38.9% with minimal clinical significance, 8.9% as definitely clinically significant and could cause patient harm while one issue (0.2%) was classified as life threatening. The main causes of PCIs were deterioration of disease state which led to failure of therapy, and also presentation of new symptoms or indications. Of the 338 PCIs where changes were recommended by the pharmacist, 87.3% were carried out as recommended.

**Conclusions:**

This study demonstrates the importance of pharmacists working in collaboration with other healthcare providers especially the medical doctors in identifying and resolving pharmaceutical care issues to provide optimal care for patients with chronic diseases.

## Background

The roles of pharmacists have evolved from product oriented, dispensing of medications to more patient-focused services such as the provision of pharmaceutical care, which includes the identification, prevention and resolution of drug-related problems (DRPs). The term “pharmaceutical care” was defined by Hepler and Strand [1]. Basically, it is the responsible provision of drug therapy by the collaboration of a clinical pharmacist with the patient as well as other members of the healthcare team in designing, implementing and monitoring a therapeutic plan that will produce specific outcomes.

DRPs are defined as “problems in the pharmacotherapy of the individual patient that actually or potentially interfere with desired health outcomes” [2]. Among the most common DRPs are: adverse drug reactions, drug choice problem, dosing problem, drug-use problem and interactions [3]. Other terminology such as pharmaceutical care issues (PCIs) has also been used [4].

Studies have shown that the cost associated with DRPs far exceed the cost of medications. Ernst & Grizzle [5] found that the estimated cost of morbidity and mortality due to DRPs was more than USD177.4 billion yearly.

Wermeille and colleagues [6] reported PCIs resolved by community pharmacists in collaboration with medical general practitioners (GPs). Other studies reported a significant reduction in HbA_1c_ in community-based patients with diabetes provided pharmaceutical care by a pharmacist [7-12]. A systematic review conducted by Royal and colleagues [13] showed that pharmacist-initiated medication review was effective in reducing hospital admission by 36%. However, most of the studies on pharmaceutical care were conducted in countries such as Australia, the United Kingdom and the United States [6,7,14-20]. Studies in South East Asia are scarce and therefore, this study is warranted to fill the information gap by identifying the types of PCIs encountered by primary care patients with diabetes mellitus, hypertension or hyperlipidaemia in Malaysia.

## Methods

### Study population

This study was part of a large controlled trial called the Cardiovascular Risk Factors Intervention Strategies (CORFIS) trial. The CORFIS trial was a community-based, multicentre trial which compared the impact of collaborative intervention by various healthcare professionals (GPs, pharmacists, dietitians and nurses) to usual standard care on patients with diabetes, hypertension or hyperlipidaemia. All the patients in the intervention group of the CORFIS trial were included in this part of the study. Patients in the control group were not included in this part of the study because under the usual healthcare practice in Malaysia, patients who seek treatment in GP clinics obtained their medications from the clinic itself and do not have to see a pharmacist. The study was approved by the Medical Research Ethics Committee of the Ministry of Health Malaysia.

Participants were recruited from 44 GP clinics in the Klang Valley, Malaysia from January to June 2008. The Klang Valley encompasses Kuala Lumpur and its vicinity which constitutes the main commercial and administrative centre in Malaysia. Inclusion criteria of patients were: participants aged 18 and above, with at least one of the following diseases: diabetes mellitus, hypertension or hyperlipidaemia, and currently managed with at least one medication. Exclusion criteria were those who did not provide written consent, were pregnant or breast-feeding, had history of unstable angina, heart failure, acute myocardial infarction, clinically significant valvular heart disease, stroke, coronary revascularization procedure, or who had serum creatinine of more than 150 mmol/L in the preceeding 6 months, or were not able to visit the clinics for monthly follow-up sessions. Written informed consent for participation in the study was obtained from all participants.

### Study procedures

**Figure 1:**
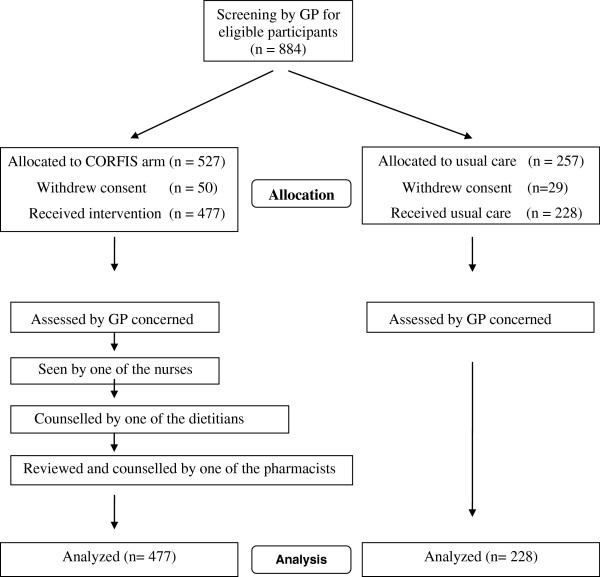
Flow chart of the CORFIS study.

The study involved a group of pharmacists, dietitians and nurses. Each participant was interviewed and counselled by one of the pharmacists, dietitians and nurses at the GP clinic where the participant was recruited (Figure 1). Each participant spent about 30 to 60 min with each of the healthcare providers. In this study, the term PCIs was used instead of DRPs to cover a wider spectrum of issues and also because pharmaceutical care was provided by service pharmacists to the participants. During the 24-week follow-up assessments, the pharmacist reviewed the participants’ medications and counselled the participants every 4 weeks, noted any PCIs encountered by the participants and helped to resolve the PCIs. If required, the pharmacist would contact the GP concerned to alert, discuss and if possible, to resolve the PCIs which could affect the participants’ clinical outcomes. Whereas, the dietitians provided dietary advice while the nurses advised the patients on general healthcare such as foot care.

### Outcome measures

The primary outcome was the types of PCIs encountered by the participants, as identified by the pharmacists. The secondary outcomes were the causes and clinical significance of the PCIs, and outcome of the interventions made by the pharmacists.

#### Data analysis

All data collected were analysed using the Statistical Package for Social Sciences (SPSS Inc., Chicago, IL, USA), version 16. Descriptive statistics were obtained for all data. Sample means and standard deviations (SDs) were presented for continuous variables while frequencies and percentages were calculated for categorical variables. All outcome measures were classified by two pharmacists who were involved with the design and conduct of the study hence, were very familiar with the PCIs identified. However, to prevent bias, the clinical significance of the PCIs was classified independently by a clinician and another pharmacist who were not involved in the study. All classifications were based on “The PCNE Classification V 5.01” [3] except for the outcome of interventions and the clinical significance of the PCIs. Some additional categories of classification were added to cover the broad spectrum of PCIs encountered in this study as some could not be put under the categories specified by the PCNE.

Clinical significance of the PCIs was classified based on the four categories used by Stubbs et al. [21]: Grade (1) probably clinically insignificant, (2) minimal clinical significance, (3) definitely clinically significant and could cause patient harm and (4) potentially life-threatening. These PCIs were initially classified independently by a clinician and a pharmacist. The results were tested for correlation using Kappa statistics, κ. The strength of agreement is as followed: κ < 0.20 is poor, 0.21–0.40 is fair, 0.41-0.60 is moderate, 0.61-0.80 is good and 0.81-1.00 is very good [22]. A consensus on the final classification was derived with the assistance of another clinician and another pharmacist.

## Results

A total of 477 participants in the CORFIS arm were included in this part of the study. The demographic data and baseline characteristics of these participants are shown in Table 1. Of the 477 participants, 268 (56.2%) had diabetes mellitus, 320 (67.1%) had hypertension, and 299 (62.9%) had hyperlipidaemia. These three chronic diseases often occurred together, with 311 participants (65.2%) who had either diabetes with hypertension, diabetes with hyperlipidaemia, hypertension with hyperlipidaemia or all the three diseases.

**Table 1:** Demographics and clinical characteristics of participants recruited at baseline

**Characteristics**	**Intervention (n = 477)**
Age , mean years (SD)	47.9 (9.6)
Gender, frequency (%)	
Male	287 (60.2)
Female	190 (39.8)
Ethnic Group, frequency (%)	
Malay	206 (43.2)
Chinese	124 (26.0)
Indian	130 (27.3)
Others	17 (3.6)
Education, frequency (%)	
Primary	25 (5.2)
Secondary	215 (45.1)
Tertiary	178 (37.3)
None	59 (12.3)
Cardiovascular comorbidities, frequency (%)	
Diabetes (DM)	45 (9.4)
Hypertension (HPT)	79 (16.6)
Hyperlipidaemia (HLP)	42 (8.8)
DM + HPT	54 (11.3)
DM + HLP	70 (14.7)
HPT + HLP	88 (18.4)
DM + HPT + HLP	99 (20.8)
Smoking history, frequency (%)	
Yes	74 (15.5)
No	403 (84.5)

Note: *DM* Diabetes mellitus, *HPT* Hypertension, *HLP* Hyperlipidaemia.

### Pharmaceutical Care Issues

Of the 477 participants, 53.7% (256 participants) had at least one PCI, giving a total of 706 issues. Details of the PCIs encountered by the participants are shown in Table 2.

**Table 2:** Categories of PCIs according to PCNE V5.01 classification

**No.**	**Primary domain**	**Code**	**Sub-classes**	**Frequency (%)**
1	Adverse drug reactions	P1.1	Hypoglycaemia	19 (2.7)
			Gastrointestinal disturbances	19 (2.7)
			Cough	13 (1.8)
			Muscle ache and cramps	10 (1.4)
			Dizziness	5 (0.7)
			Others	44 (6.2)
	**Subtotal**			**110 (15.6)**
2	Drug choice problems	P2.1	Inappropriate drug	6 (0.8)
		P2.3	Inappropriate duplication	5 (0.7)
		P2.4	Contraindication	1 (0.1)
		P2.5	No clear indication for drug	10 (1.4)
		P2.6	No drug prescribed but clear indication	41 (5.8)
		P2.7	Inadequate regimen	3 (0.4)
		P2.8	Drug not required	1 (0.1)
	**Subtotal**			**67 (9.5)**
3	Dosing problem	P3.1	Dose too low or frequency not enough	10 (1.4)
		P3.2	Dose too high or frequency too often	10 (1.4)
		P3.3	Duration of treatment too short	1 (0.1)
		P3.4	Duration of treatment too long	1 (0.1)
		P3.5	Inappropriate dosing	2 ((0.3)
	**Subtotal**			**24 (3.4)**
4	Drug use problems	P4.1	Drug not taken or administered at all	20 (2.8)
		P4.3	Incorrect administration	69 (9.8)
		P4.4	Non-adherence to medication	146 (20.7)
	**Subtotal**			**235 (33.3)**
5	Drug interaction	P5.1	Potential interaction	**4 (0.5)**
6	Others	P6.2	Insufficient awareness/knowledge	**144 (20.1)**
		P6.3	Unclear complaints	82 (11.6)
		P6.4	Therapy failure	**98 (13.9)**
		P6.5	Worries about complications/ADRs	15 (2.1)
		P6.6	Dispensing issues	**11 (1.6)**
		P6.7	Lifestyle modifications	8 (1.1)
		P6.8	Monitoring for side effects	3 (0.4)
		P6.9	Technical issues	2 (0.3)
	**TOTAL**			**706 (100)**

*Subclasses added, not in The PCNE Classification V 5.01.

Non-adherence to medications was the most common drug-use problem (146 of 235 issues, 62.1%). Other drug-use problems involved participants administrating their medications at incorrect doses or frequencies or incorrect timing with respect to meals. For example, metformin and aspirin were taken before meals and sulphonylureas were taken at bedtime.

PCIs classified under “insufficient awareness or knowledge” included 47 issues pertaining to the misconception of participants concerning the use of medications and participants’ lack of knowledge and queries about their medications and also their disease states. Fifteen issues were related to participants’ undue worries about the side effects and complications related to their medications and another 82 issues of unclear complaints concerning some health problems which may be related to patients’ medications were also included in this category.

Adverse drug reactions (ADRs) included side effects of medications as reported by the participants. Examples of these side effects are as shown in Table 2. Hypoglycaemia was related to the use of antidiabetic agents. Gastrointestinal disturbances included gastritis due to non-steroidal anti-inflammatory drugs (NSAIDs) and diarrhoea associated with the use of metformin (6 cases each). Another 12 participants complained of dry cough attributed to the use of angiotensin-converting enzyme inhibitors while 10 participants complained of muscle aches and cramps after using statins. ADRs classified as “Others” included dermatological reactions such as eczema, rashes and itch; weight gain; frequent urination; hair loss; bradycardia; bleeding from the anus; blocked nose and flushing.

Therapy failure was assumed when participants’ blood pressure (BP), blood glucose levels or lipid levels were not at target levels despite being on pharmacological treatment. Whereas, drug choice problem in this study involved participants who had been seen by the GPs but still with high BP, high blood glucose or high cholesterol level and were not on any medications. Inappropriate duplication of therapeutic group or active ingredient involved two participants who were given both glimepiride and gliclazide, another participant was given gliclazide 80 mg (Diamicron®) and modified-release gliclazide 30 mg (Diamicron MR®) while another participant was given irbesartan (Approvel®) and a combination of irbesartan and hydrochlorothiazide (Co-Approvel®). In addition, one participant was on both loratadine and desloratadine. The only case of contraindication encountered in this study involved a woman planning to get pregnant but was prescribed a combination of irbesartan and hydrochlorothiazide (Co-Approvel®) [23].

Dosing problems included doses and frequencies of medications prescribed which were either too high or too low. Two cases with problems in the duration of treatment involved a participant given a statin for only a week and another who was still prescribed a statin even though his cholesterol level was only 2.9 mmol/L and his low-density lipoprotein (LDL) was 1.3 mmol/L.

Dispensing issues involved incorrect labeling of the dosage or frequency of antidiabetic drugs. There were two cases where the strength of enalapril was wrongly stated on the label. Another case involved atorvastatin (Storvas®) being labeled as simvastatin. In addition, a participant was prescribed irbesartan (Approvel®) but was dispensed irbesartan and hydrochlorothiazide (Co-Approvel®). Another participant was dispensed 40 mg of atorvastatin although was prescribed only 10 mg. This subclass of PCIs also included two cases of inappropriate storage of medication. Glyceryl trinitrate (GTN) was dispensed in a plastic envelope instead of its original bottle. Atovarstatin was kept in a plastic envelope and the colour of the tablets had changed. These PCIs were brought to the attention of the GPs or clinic staff concerned to prevent repetition of such issues.

Potential drug interactions included the use of enalapril with allopurinol which may increase the risk of leucopenia and hypersensitivity induced by allopurinol; fenofibrate and atorvastatin which may increase the risk of myopathy, and the use of atorvastatin with alcohol which may increase the risk of liver problems [23,24].

The eight cases of lifestyle modifications included participants who were current smokers and were counselled on smoking cessation. There were several cases where participants asked about diet and exercise but these were referred to the dietitian and not considered as PCIs. Monitoring of side effects included alerting the doctors to monitor participants’ liver function for those on statins and blood glucose level in a patient suspected to have diabetes.

### Classification of PCIs according to clinical significance

A Cohen’s Kappa statistic (k) of 0.729 (*P* = 0.036) was obtained which implied good agreement in classification between the two assessors. Of the 706 PCIs, 52% were classified as probably clinically insignificant, 38.9% with minimal clinical significance, 8.9% as definitely clinically significant and could cause patient harm while one issue (0.2%) where a participant reported bleeding from the anus attributed to the use of aspirin was classified as life threatening.

### Causes of PCIs

Causes of PCIs are as shown in Table 3. Non-adherence to medications was attributed to forgetfulness or to participants having doubts or misconception about the purpose and effectiveness of their medications. Some participants reported that they were not clear regarding the dosage and use of their medications while others were unwilling to take their medications due to fear of side effects.

**Table 3:** Causes of PCIs (using The PCNE Classification V 5.01)

**Primary domain**	**Code V5.01**	**Cause**	**Frequency (%, n = 706)**
**1. Drug/Dose selection**	C1.1	Inappropriate drug selection	7 (1.0)
	C1.2	Inappropriate dosage selection	10 (1.4)
	C1.3	More cost-effective drug available	2 (0.3)
	C1.4	Pharmacokinetic problems, include ageing/deterioration in organ function and interactions	5 (0.7)
	C1.5	Synergistic/preventive drug required and not given	11 (1.6)
	C1.6	Deterioration/improvement of disease state	150 (21.2)
	C1.7	New symptom or indication revealed/presented	75 (10.6)
	C1.8	Manifest side effect, no other cause	93 (13.2)
		**Subtotal 1**	**353 (50.0)**
**2. Drug use process**	C2.1	Inappropriate timing of administration and/or dosing intervals	44 (6.2)
	C2.2	Drug underused/ under-administered	5 (0.7)
	C2.3	Drug overused/ over-administered	9 (1.3)
	C2.6	Patient unable to use drug/form as directed	2 (0.3)
	C2.7	Too many medications*	6 (0.8)
	C2.8	Inappropriate duration of use*	1 (0.1)
	C2.9	New drug prescribed*	2 (0.3)
		**Subtotal 2**	**69 (9.8)**
**3. Information**	C3.1	Instructions for use/taking not known	2 (0.3)
	C3.2	Patient unaware of reason for drug treatment	16 (2.3)
	C3.5	Lack of communication between HCPs	1 (0.1)
	C3.6	Miscommunication between doctor and patient*	4 (0.6)
	C3.7	Unclear label*	6 (0.8)
		**Subtotal 3**	**29 (4.1)**
**4. Patient/Psychological**	C4.1	Patient forgets to use/take drug	34 (4.8)
	C4.2	Patient has concerns with drugs	24 (3.4)
	C4.3	Patient suspects side effects	37 (5.2)
	C4.4	Patient unwilling to carry financial cost	6 (0.8)
	C4.7	Patient unwilling to adapt life-style	3 (0.4)
	C4.8	Burden of therapy	3 (0.4)
	C4.9	Treatment not in line with health beliefs	6 (0.8)
	C4.11	Religious belief*	6 (0.8)
	C4.12	Patient unaware of possible complications*	8 (1.1)
	C4.13	Patient unaware of drug name*	5 (0.7)
	C4.14	Out of medication*	7 (1.0)
	C4.15	Overseas or travelling*	9 (1.3)
	C4.16	Patient smoking*	7 (1.0)
	C4.17	Patient unaware of tablet strength*	1 (0.1)
	C4.18	Patient does not trust HCPs*	1 (0.1)
	C4.19	Patient on traditional medicine*	1 (0.1)
	C4.20	Patient on too many supplements*	3 (0.4)
	C4.21	Patient’s meal time is different*	1 (0.1)
	C4.22	Patient shared medications	1 (0.1)
	C4.23	Non-adherence to medications*	65 (9.2)
		**Subtotal 4**	**228 (32.3)**
**5. Logistics**	C5.2	Prescribing error (only in case of slip of the pen)	1 (0.1)
	C5.3	Dispensing error (wrong drug or dose dispensed)	1 (0.1)
		**Subtotal 5**	**2 (0.3)**
**6. Others**	C6.11	Patient anxious about glucose level*	1 (0.1)
	C6.12	Patient not sure of using glucose meter or other devices*	2 (0.3)
	C6.13	Wrong frequency of glucose monitoring*	1(0.1)
	C6.14	Drug stored in unsuitable condition*	2 (0.3)
	C6.15	Patient not on medication in record*	3 (0.4)
	C6.2	No obvious cause	16 (2.3)
		**Subtotal 6**	**25 (3.5)**

*Subclasses added, not in The PCNE Classification V 5.01.

HCPs = Healthcare professionals.

### Outcome of pharmacist intervention

The most common intervention made by the pharmacists was the counselling of patients on their medications (38.8%), followed by the referral of patients to the prescribers (20.8%), educating patients concerning their disease states (12.0%), recommending a change in the dose or frequency of the medications or to add another medication (5.8%) and to monitor the patient’s condition (5.0%).

The outcome of pharmacist intervention is as shown in Table 4. If the PCIs with “unknown outcomes” and “no change required” categories were excluded, then 87.3% (295 out of 338) of changes were made as recommended by the pharmacist. No change was made when the prescriber or patient preferred to continue monitoring the patient’s condition or to wait until the next clinic appointment before making any changes. Majority of pharmacist interventions were directed at the patients (496 of 702; 70.9%) with only 29.1% (204 of 702) that involved the medical doctors. One of the PCIs required action to be taken by the caregiver and another by the clinic staff. If “unknown outcomes” and “no change required” were excluded, 77.9% of changes recommended by the pharmacists were accepted by the doctors. However, 91.5% of changes recommended by the pharmacists were carried out by the patients.

**Table 4:** Outcome of interventions

**Outcome of interventions**	**Frequency (% of subtotal)**		Frequency (%, n = 702*)
	**GP**	**Patient**	**Overall**
Change made as per pharmacist recommendation	81 (77.9)	214 (91.5)	295 (42.0)
Change made not as per pharmacist recommendation	1 (1.0)	2 (0.9)	3 (0.4)
No change made, medication not dispensed	1 (1.0)	0 (0)	1 (0.1)
No change made	21 (20.2)	18 (7.7)	39 (5.6)
**Subtotal**	**104**	**234**	**338**
No change required	11	100	111 (15.7)
Outcome unknown	89	162	251 (36.2)
**Total**	**204**	**496**	**700**

Note: Four of the PCIs did not mention the action taken by the pharmacist to resolve the issues and hence the total PCIs in Table 4 is only 702*. However, the total number of PCIs which involved the GPs and the patients was only 700 as two of the PCIs which required action by the clinic staff or the caregiver are not shown in Table 4.

## Discussion

This study is probably the first large scale trial conducted in Malaysia which involved collaboration between various healthcare professionals in the management of diabetes, hypertension or hyperlipidaemia at primary care level. More than half of the participants encountered at least one PCI, with a total of 706 PCIs identified.

Drug-use problems (especially non-adherence to medication), ADRs, therapeutic failure and drug-choice problems constituted the main PCIs encountered by participants in this study, followed by insufficient awareness or knowledge of participants. Wermeille and colleagues [6] reported similar results. The high number of PCIs encountered by the participants indicates the importance of pharmacists working in collaboration with other healthcare providers to identify and resolve such problems. These include educating participants on the purpose and side effects of medications to clear their doubts and misconception, which would lead to better understanding and hence better medication adherence [25].

Participants were taking their medications incorrectly in terms of dose, frequency and timing in relation to meals (8.2% of the PCIs). This again indicates that the provision of pharmaceutical care is essential to identify and resolve such problems in order to achieve optimal clinical outcomes and also to reduce side effects such as gastrointestinal disturbances.

Therapy failure constitutes 13.9% of the PCIs. The usual recommendations for such issues were to increase the dose or frequency of existing medication or to add another medication. Patients were also advised to monitor their BP or blood glucose level where appropriate. Constant monitoring of BP, blood glucose levels and lipid profile is crucial to ensure that these clinical conditions are within target levels in order to prevent complications and to reduce morbidity and mortality [26,27]. In addition, lifestyle modification was also often recommended.

The 11 dispensing issues identified in this study are not reflective of the incidence of dispensing problems encountered as this study was not designed specifically to determine dispensing errors, hence no direct observation of the dispensing process was carried out. Examples of dispensing errors noted in this study only served to alert the healthcare professionals, especially those involved in dispensing of medications at the primary care clinics, that such errors may occur and measures should be taken to minimize such risk.

Drug choice and dosing problems are usually detected via double-checking by an independent person. Pharmacists can act as a safety net to prevent or minimize any potential medication errors. These included a case of contraindication which involved a woman planning to get pregnant but was prescribed an angiotensin-receptor blocker which carries a potential risk of teratogenesis if the woman did get pregnant [23]. Again, the number of prescribing discrepancies that were identified is not indicative of the incidence of such problems as prescriptions issued to participants of this study were not screened individually.

Most of the PCIs were considered to be of no direct potential clinical significance (52%), especially non-adherence to medications and some minor side effects, but they could cause inconvenience and prolongation of the issues may lead to complications and increased cost of treatment. Incorrect timing of drug administration were considered to have minimal clinical significance, except for aspirin which has a higher risk of causing gastrointestinal complications and thus was classified as definitely clinically significant.

The main causes of PCIs were deterioration or improvement of disease state which led to therapy failure, and also presentation of new symptoms or indication. This indicates the role of pharmacists in monitoring patients with chronic diseases. The manifestation of side effects such as cough, gastrointestinal problems and symptoms of hypoglycaemia as well as patients’ concerns with drugs and undue worries about side effects were also common causes of PCIs. In these aspects, counselling of patients by the pharmacist is important to resolve some of the preventable side effects and also to assure and increase patients’ confidence concerning their medications. Consequently, educating and counselling patients on their medications and disease states were the main interventions made by the pharmacists (50.8%) in this study. Often, patients were referred to the prescribers (20.8%) especially if adjustment to patients’ prescribed medication regimens were deemed necessary.

In this study, most of the recommendations made by the pharmacists (87.3%) were carried out accordingly. This indicates the effectiveness of the pharmacist interventions in resolving PCIs as well as the doctor and patients’ confidence in following the recommendations made by pharmacists. Other studies have also shown that pharmacist interventions produced positive outcomes [13,28,29] and well accepted by the doctors and patients [6].

This study has several strengths. The CORFIS trial was performed in private primary care settings in Malaysia, highlighting the feasibility of collaboration between pharmacists, GPs, dietitians, nurses and patients in diabetes, hypertension and hyperlipidaemia risk management. The study had an adequate sample size and follow-up duration. It is probably the first study in Malaysia, which involved the collaboration of various healthcare professionals in managing primary care patients with chronic diseases.

There are also several limitations in this study. The process of detecting and resolving PCIs is very time consuming since there is a time-lag between identification and subsequently communicating the PCI to the caregiver involved, especially in a community setting. Potential bias in the detection and resolving of PCIs may exist since this depended heavily on the experience of the pharmacist performing the medication review. The classification of the PCIs, causes and outcomes were performed by two pharmacists based on the information recorded by the service pharmacist. Although care has been taken to be as accurate as possible in the classification, some ambiguities could not be ruled out. In addition, the classification of the clinical significance of the PCIs identified was not re-tested.

## Conclusions

This study identifies the types of PCIs encountered by patients with diabetes, hypertension or hyperlipidaemia. It also demonstrates the importance of pharmacists working in collaboration with other healthcare providers especially the GPs, in resolving these PCIs to provide optimal care for patients with chronic diseases.

## Abbreviations

PCI: Pharmaceutical care issue; DRP: Drug-related problem; ADR: Adverse drug reaction; GP: Medical general practitioner; HbA_1c_: Glycosylated haemoglobin; PCNE: Pharmaceutical Care Network Europe; CORFIS: Cardiovascular Risk Factors Intervention Strategies.

## Competing interests

The authors declare that they have no competing interests.

## Authors’ contributions

SSC is the head of the Pharmacist Research Group in the CORFIS trial, and was involved in the initiation, planning, development, implementation and coordination of the project, data analysis and also drafting of the manuscript. LCK was involved in the coordination of the project, entered and analysed the data, and also helped to draft the manuscript. FAMY was involved in the initiation, planning, development, implementation and coordination of the project. GHT was involved in the coordination of the project and also in data collection and analysis. SLWH contributed to the data analysis and drafting of the manuscript. BE and TP were involved in the planning, development, implementation and coordination of the project. All authors checked and approved the final manuscript.

## Pre-publication history

The pre-publication history for this paper can be accessed here:

http://www.biomedcentral.com/1472-6963/12/388/prepub

## References

[B1] Hepler C Strand L Opportunities and responsibilities in pharmaceutical care Am J Hosp Pharm 1990 47 533 543 2316538

[B2] van Mil F Drug-related problems: a cornerstone for pharmaceutical care J Malta College Pharm Pract 2005 10 5 8

[B3] Pharmaceutical Care Network Europe (PCNE) Foundation PCNE classification for drug related problems(revised 01-05-06 vm) V5.01; 2006 http://www.pcne.org/sig/drp/documents/PCNE%20classification%20V5.01.pdf

[B4] Krska J Jamieson D Arris F McGuire A Abbott S Hansford D A classification system for issues identified in pharmaceutical care practice Int J Pharm Pract 2002 10 91 100 10.1111/j.2042-7174.2002.tb00593.x

[B5] Ernst FR Grizzle AJ Drug-related morbidity and mortality: updating the cost-of-illness model J Am Pharm Assoc 2001 41: 192 199 10.1016/s1086-5802(16)31229-3 11297331

[B6] Wermeille J Bennie M Brown I McKnight J Pharmaceutical care model for patients with type 2 diabetes: integration of the community pharmacist into the diabetes team: a pilot study Pharm World Sc 2004 26 18 25 15018255 10.1023/b:phar.0000013465.24857.a8

[B7] Davis TM Clifford RM Davis WA Batty KT The role of pharmaceutical care in diabetes management Brit J Diabet Vasc Dis 2005 5 352 356 10.1177/14746514050050061001

[B8] Borges A Guidoni C Ferreira L Freitas OD Pereira L The Pharmaceutical care of patients with type 2 diabetes mellitus Pharm World Sc 2010 32 6 730 736 10.1007/s11096-010-9428-3 20734138

[B9] Jameson JP Baty PJ Pharmacist collaborative management of poorly controlled diabetes mellitus: a randomized controlled trial Am J Manag Care 2010 16 4 250 255 20394460

[B10] Krass I Armour CL Mitchell B Brillant M Dienaar R Hughes J Pharmacy diabetes care program: assessment of a community pharmacy diabetes service model in Australia Diabet Med 2007 24 677 683 10.1111/j.1464-5491.2007.02143.x 17523968

[B11] Mehuys E Van Bortel L De Bolle L Van Tongelen I Annemans L Remon JP Effectiveness of a community pharmacist intervention in diabetes care: a randomized controlled trial J Clin Pharm Ther 2011 36 5 602 613 10.1111/j.1365-2710.2010.01218.x 21143256

[B12] Scott DM Boyd ST Stephan M Augustine SC Reardon TP Outcomes of pharmacist-managed diabetes care service in a community health centre Am J Health Syst Pharm 2006 63 2116 2122 10.2146/ajhp060040 17057049

[B13] Royal S Smeaton L Avery A Hurwitz B Sheik A Interventions in primary care to reduce medication related adverse events and hospital admissions: systematic review and meta analysis Qual Saf Health Care 2006 15 23 31 10.1136/qshc.2004.012153 16456206 PMC2563996

[B14] Armour CL Taylor SJ Hourihan F Smith C Krass I Implementation and evaluation of Australian pharmacists’ diabetes care services J Am Pharm Assoc 2004 44 456 466 10.1331/1544345041475625 15372866

[B15] Choe HM Mitrovich S Dubay D Hayward RA Krein SL Vijan S Proactive case management of high-risk patients with type 2 diabetes mellitus by a clinical pharmacist: a randomized controlled trial Am J Manag Care 2005 11 253 260 15839185

[B16] Clifford RM Batty KT Davis TME Davis W Stein G Stewart G A randomised controlled trial of a pharmaceutical care programme in high-risk diabetic patients in an outpatient clinic Int J Pharm Pract 2002 10 85 89 10.1111/j.2042-7174.2002.tb00592.x

[B17] Davidson MB Karlan VJ Hair TL Effect of a pharmacist-managed diabetes care program in a free medical clinic Am J Med Qual 2000 15 137 142 10.1177/106286060001500403 10948785

[B18] Irons BK Lenz RJ Anderson SL Wharton B Habeger B Anderson G Retrospective cohort analysis of the clinical effectiveness of a physician-pharmacist collaborative drug therapy management diabetes clinic Pharmacotherapy 2002 22 1294 1300 10.1592/phco.22.15.1294.33476 12389879

[B19] Jaber L Halapy H Fernet M Tummalapalli S Diwakaran H Evaluation of a pharmaceutical care model on diabetes management Ann Pharmacother 1996 30 238 243 8833557 10.1177/106002809603000305

[B20] Rothman RL Malone R Bryan B A randomized trial of a primary care-based disease management program to improve cardiovascular risk factors and glycated hemoglobin levels in patients with diabetes Am J Med 2005 118 276 284 10.1016/j.amjmed.2004.09.017 15745726

[B21] Stubbs J Haw C Cahill C Auditing prescribing errors in a psychiatric hospital. Are pharmacists’ interventions effective? Hosp Pharm 2004 11 203 206

[B22] Altman DG Practical Statistics for Medical Research 1999 1 United States: Chapman & Hall/CRC

[B23] The Joint Formulary Committee British National Formulary (BNF) 2012 63 London: BMJ Group and Pharmaceutical Press

[B24] MICROMEDEX® 1.0 Drug Interactions Thomson Reuters (Healthcare) Inc http://www.micromedex.com. (accessed February 20, 2010)

[B25] Lee JK Grace KA Taylor AJ Effect of a pharmacy care program on medication adherence and persistence, blood pressure, and low-density lipoprotein cholesterol: A randomized controlled trial JAMA 2006 296 2563 2571 10.1001/jama.296.21.joc60162 17101639

[B26] Gæde P Lund-Andersen H Parving HH Pedersen O Effect of a multifactorial intervention on mortality in type 2 diabetes N Engl J Med 2008 358 580 591 10.1056/NEJMoa0706245 18256393

[B27] UK Prospective Diabetes Study (UKPDS) Group Tight blood pressure control and risk of macrovascular and microvascular complications in type 2 diabetes: UKPDS 38 BMJ 1998 317 703 713 10.1136/bmj.317.7160.703 9732337 PMC28659

[B28] McLean DL McAlister FA Johnson JA King KM Makowsky MJ Jones CA A randomized trial of the effect of community pharmacist and nurse care on improving blood pressure management in patients with diabetes mellitus: study of cardiovascular risk intervention by pharmacists-hypertension (SCRIP-HTN) Arch Intern Med 2008 168 2355 2361 10.1001/archinte.168.21.2355 19029501

[B29] Cohen LB Taveira TH Khatana SAM Dooley AG Pirraglia PA Wu W-C Pharmacist-led shared medical appointments for multiple cardiovascular risk reduction in patients with type 2 diabetes Diabetes Educ 2011 37 6 801 812 10.1177/0145721711423980 22021025

